# DC Magnetron Sputtering for Synthesizing Bimetallic NiFe Thin Films as Efficient OER-Catalyzing Electrodes

**DOI:** 10.3390/ma19153155

**Published:** 2026-07-23

**Authors:** Daniyal Hasan, Albano Cavaleiro, Diogo Cavaleiro, Jose David Castro, Jolanta Ewa Klemberg-Sapieha, Eduardo Silva, Sandra Carvalho

**Affiliations:** 1 Centre for Mechanical Engineering, Materials and Processes (CEMMPRE), Department of Mechanical Engineering, University of Coimbra, 3030-788 Coimbra, Portugal; 2 IPN—LED&MAT—Instituto Pedro Nunes, Laboratório de Ensaios, Desgaste e Materiais, Rua Pedro Nunes, 3030-199 Coimbra, Portugal; 3 Department of Engineering Physics, Polytechnique Montréal, Montreal, QC H3T 1J4, Canada; 4 Durit Coatings, Parque Industrial de Taveiro n° 41 e 42, 3045-508 Coimbra, Portugal

**Keywords:** DC magnetron sputtering, nickel-iron, oxygen evolution reaction, electrocatalyst, thin-film electrodes

## Abstract

Industrial water electrolysis systems for hydrogen production use noble metal-based RuO_2_/IrO_2_ to catalyze the oxygen evolution reaction (OER). Replacing noble metal compounds with earth-abundant NiFe compounds requires a scalable synthesis technique with structural and morphological control. In this study, DC magnetron sputtering was systematically investigated as a scalable approach for synthesizing NiFe bimetallic thin-film electrocatalysts as alternatives to noble metal-based materials. NiFe thin films (~200 nm) with varying Fe contents were deposited on SS-316L substrates and characterized using compositional, morphological, structural, and electrochemical techniques. Scanning electron microscopy revealed a columnar morphology, while X-ray diffraction confirmed the formation of a NiFe random solid solution. The Fe ratio was found to strongly influence OER performance, with the Fe_24_Ni_76_ composition exhibiting the best activity, requiring an overpotential of 361 mV vs. RHE to achieve 10 mA · cm^−2^ and delivering a current density of 363 mA · cm^−2^ at 1.7 V vs. RHE. X-ray photoelectron spectroscopy indicated that the enhanced activity of Fe_24_Ni_76_ originated from increased oxidation of metallic Ni and a higher density of catalytically active oxide species. These results demonstrate that sputtered NiFe thin films, particularly at optimized compositions, are promising scalable and low-cost OER electrodes for next-generation water electrolysis systems.

## 1. Introduction

In pursuance of the Sustainable Development Goal (SDG) 7 on clean and affordable energy, hydrogen has emerged as a key player [1] owing to its applications in fuel cells [2], metal-air batteries [3], and most importantly as a storage medium for sustainable resources of energy [4]. Sustainable resources like solar and wind energy are not consistent throughout the year and not even throughout the day. Surplus electrical energy from sustainable resources can be used for water electrolysis, and the produced hydrogen can be used as a consistent source of energy [5]. Hydrogen has a high energy density and can effectively store electrical energy in the form of chemical energy [6,7,8,9]. The whole process of producing and consuming hydrogen has a zero-carbon footprint.

The process of hydrogen production through water splitting or electrolysis has two half-cell reactions: the hydrogen evolution reaction (HER) occurring at the cathode and the oxygen evolution reaction (OER) occurring at the anode [10]. OER occurring at the anode is the rate-limiting step [11,12] as it has a complicated chemistry involving transfer of four electrons, thus making OER kinetically sluggish [13,14]. OER is traditionally catalyzed by noble metal-based compounds, like RuO_2_/IrO_2_ [15]. High cost and scarcity of noble metals increase the overall cost of the process and make the water electrolysis process economically unviable for large-scale applications. Thus, optimization of OER is crucial for the viability of the water electrolysis process [16]. It requires an OER-catalyzing anode material, which is cost-effective, has fast reaction kinetics, and is synthesized by an industrially scalable technique.

Transition metals have variable oxidation states and are able to electrocatalyze reversible oxidation-reduction steps involved in OER [17,18]. Among these, Ni- and Fe-based materials (abundant, low cost, and good catalytic activity) have attracted considerable research interest [12,18,19]. A wide range of Ni- and Fe-containing compounds have been explored, including their alloys [20], oxides [19], hydroxides [21], phosphides [22], sulphides [23], chalcogenides [24], single-atom catalysts [25], and metal–organic frameworks [26].

Furthermore, the combination of Fe and Ni as bimetallic compounds/complexes has been shown to have even better OER catalytic performance than their singular counterparts [27,28]. The addition of Fe in Ni creates a coupling effect called the “Fe effect” [29]. Fe dissolves into the Ni oxide matrix, and the local electronic coordination favors OER by lowering the onset potential of OER. Effectively arranging the active sites by dispersing Fe in the Ni matrix to work together in a synergistic way is important and remains a challenge [30]. OER performance of electrodes is also dependent upon morphological factors like surface area, structure, and grain size [21,31,32,33,34,35]. Thus, to achieve the desired morphology and optimum arrangement of active sites, different synthesis routes, typically wet chemical synthesis, have been used, as summarized in [29]. Electrode synthesis techniques should also be scalable to meet industrial demands.

Magnetron Sputtering is a physical vapor deposition (PVD) technique that enables the fabrication of thin films with carefully tailored structures and morphologies [36] and is well known for its versatility in depositing a wide range of materials [37]. Thin film depositions have minimum bulk electrical resistance, have columnar morphology, which provides more surface area, and atoms are well dispersed; thus, better local electronic coordination takes place for maximum synergistic effect.

In this context, this study focused on using DC magnetron sputtering as a scalable technique to synthesize NiFe bimetallic compositions in the form of thin films. NiFe thin-film compositions were deposited over SS-316L conductive substrates. Electrochemical testing of the films was conducted to verify the effectiveness of sputtered thin films as efficient OER-catalyzing electrodes. Stability of electrochemical performance was also tested. NiFe bimetallic materials synthesized from different processes report optimal compositions between 20 and 40 atomic percent Fe in Ni [11,27,38,39,40]; thus, five different compositions were deposited to find the optimum NiFe composition for sputtered thin-film electrodes. Unconventional NiFe-based thin-film electrodes are many times cheaper in price and require less material, yet they demonstrated efficient OER catalytic performance and were comparable with conventionally synthesized noble metal-based electrodes by every standard.

## 2. Materials and Methods

### 2.1. Substrate Preparation

Five sets of thin films with increasing Fe contents were deposited, which were labeled as Fe_X_Ni_1−X_, where x was the atomic percentage of Fe. Thin films were deposited over (111) Silicon wafers (Seyang Electronics Co., Ltd., Gunpo-si, Gyeonggi-do, Republic of Korea) and 20 × 20 × 1 mm^3^ SS-316L substrates. Silicon wafers were used for compositional, morphological and structural characterizations, while steel substrates were used to conduct electrochemical tests. The SS-316L substrates were given a close-to-mirror-like finish, polished sequentially with sandpapers of grit sizes P1000 and P2500 and eventually with a 3 µm diamond paste (DUOPAT-M, metkon Instruments Inc., Bursa, Turkey) [41]. Before mounting in the deposition chamber, all the substrates were ultrasonically cleaned in ethanol and acetone for 10 min each.

### 2.2. Deposition Parameters

The deposition of the coatings was conducted by DC magnetron sputtering in a nanoclusters coating deposition chamber using a pure Ni target (200 × 100 × 1 mm^3^ with a 99.7% purity from Photon Export, Barcelona, Spain) and an Invar target, which is an alloy with 64 wt.% Fe and 36 wt.% Ni, (200 × 100 × 1 mm^3^ with a 99.95% purity from Photon Export, Barcelona, Spain). All the depositions were conducted by keeping a constant power of 1000 W applied to the Invar target and by varying the power applied to the Ni target from 500 W to 1200 W to achieve the different Fe contents. Before the depositions, the chamber was evacuated to a pressure of 6 × 10^−4^ Pa. Then, cleaning of the targets and substrates through plasma etching was conducted by applying a power of 200 W to both the targets and keeping a bias potential of 600 V at the substrate, with an Ar (95% volume and balance Hydrogen from Nippon Gases, Madrid, Spain) pressure of 3 Pa, while keeping shutters in front of the targets to avoid cross-contamination of the substrate. The depositions of all the films were conducted at a 10 Pa working pressure. The substrate holder was positioned at the center of the chamber, rotating constantly at 22 rpm.

### 2.3. Compositional, Morphological and Structural Characterization Techniques

Surface/cross-sectional morphology and film thicknesses were studied using scanning electron microscopy (SEM, Zeiss Merlin Gemini 2, Jena, Germany). Chemical composition was measured using Energy-dispersive X-ray Spectroscopy (EDS, Oxford Instruments, High Wycombe, Oxford, UK, X-MAXN) at 5 kV excitation energy. Surface roughness of the thin films was studied by atomic force microscopy (AFM, Innova, Bruker, Billerica, MA, USA) in tapping mode. A silicon nitride tip with less than 8 nm radius was used. AFM micrographs were drawn over surface areas of 2 × 2 μm^2^. The structure of the films was analyzed by X-ray diffraction (XRD) using a PANalytical Phillips X’pert equipment (Malvern, UK) with Co Kα radiation (λ = 0.179 nm) at 45 kV and 40 mA using a step size of 0.025°.

Chemical structure was determined by X-ray photoelectron spectroscopy (XPS—Thermo Scientific ESCALAB Xi, Waltham, MA, USA). Spectra were obtained using monochromatic Al Kα radiation (1486.6 eV) as an X-ray excitation source and high-resolution spectra were acquired at 20 eV pass energy. To prevent surface modification or preferential sputtering of elements, argon cluster ions with an energy of 4.0 keV and a cluster size of 500 atoms were used for 200s to remove adsorbed species and organic contamination. All obtained spectra were post-processed in the Avantage v6.5 software; samples were grounded and did not require any charge compensation.

### 2.4. Electrochemical Characterization

Electrochemical tests were performed using a Gamry 600 Potentiostat (Warminster, PA, USA) connected to an electrochemical cell (three-electrode configuration). A 1 M KOH (85% purity, AR Grade, Fisher Scientific, Loughborough, UK) solution (pH = 13.6) was employed as the electrolyte. The coated SS-316L coupons (exposed area = 0.28 cm^2^), a saturated Ag/AgCl electrode, and a graphite electrode were used as working, reference, and counter electrodes, respectively. The cell was naturally aerated, and measurements were conducted at room temperature without electrolyte agitation. All the tests were performed three times after achieving electrochemical stabilization at open-circuit potential (OCP) for 3600 s. The EIS data fitting was performed in the GAMRY Echem 2 software (Version 7.10.3.14563).

Linear sweep voltammetry (LSV) was performed at a scan rate of 1 mV · s^−1^ in the potential range of 0 to 1.7 V vs. RHE. Polarization curves were subjected to 90% iR-compensation, which was performed using the formula

$$E_{iR-compensated}=E_{RHE}-{(R}_{s}\times i\times 0.90)$$

where *R_s_* is solution resistance in ohms and i is current in amperes. The solution resistance (*R_s_*) was measured in the above-mentioned potentiostat. Tafel slopes were calculated in the linear portion of the 90% iR-corrected LSV scans by plotting the logarithmic value of current against the voltage.

Cyclic voltammetry (CV) was performed in the non-faradic region in the scan range of −0.17 to −0.07 V vs. Ag/AgCl at scan rates of 20, 40, 60, 80, and 100 mV · s^−1^ with ten cycles for each scan to ensure stability of response. Double-layer capacitances (*C_dl_*) were calculated through CV scans. *C_dl_* was calculated by plotting current Δj (ja − jc) against scan rate. Roughness factor (*RF*) was calculated using the formula

$$RF=C_{dl}/C_{s}$$

where *C_s_* is the capacitance of a flat metal electrode in an aqueous KOH solution, which is reported as 0.04 mF · cm^−2^ [42]. Electrochemically active surface area (ECSA) was calculated using the formula

$$ECSA=\frac{C_{dl}}{C_{s}\times A_{geometric}}.$$

The experimental data were converted to RHE reference using the equation $E_{RHE}=E_{Ag/AgCl}+0.059pH+0.197$ [43], where 0.197 V is the reference electrode potential for Ag/AgCl. The potential values recorded during an experiment are also dependent on the molarity and pH of the electrolyte [21,44]. The conversion of experimental potential values to RHE reference not only provided a standard reference but also accommodated the factors of pH and molarity of the electrolyte.

Electrochemical impedance spectroscopy (EIS) was performed in the non-Faradic region by applying 0.45 V vs. Ag/AgCl DC potential with 5 mV rms and within the frequency range from 10^5^ to 10^−1^ Hz.

Chronopotentiometry tests were performed to check the stability in electrode performance. A current density of 10 mA · cm^−2^ was maintained for 48 h, and then on the same sample, the current density was increased up to 100 mA · cm^−2^ and maintained for 12 h. To evaluate stability at high current densities, a constant 300 mA · cm^−2^ current density was kept for 20 h on a new sample, and any changes in the overpotential required to maintain this current density were monitored over time.

## 3. Results and Discussion

### 3.1. Sputtering of Ni-Fe Magnetic Materials

Magnetron sputtering enhances plasma generation by applying a magnetic field through electromagnets at the back side of targets. Ni and Fe are ferromagnetic materials [45]; thus, the effect of the applied magnetic field is lost. It is difficult to sputter ferromagnetic materials; it was achieved by adjusting various factors. Instead of the pure Fe target, Invar, an alloy of Fe and Ni, was used, which has less magnetic strength. Ni and Invar targets with minimum thicknesses (1 mm) were procured. Normally, sputtering is performed at low Ar pressures, but here, due to the ferromagnetic nature of the material at low pressures, there were not enough ions to generate and sustain plasma. High Ar pressures were used for target cleaning (3 Pa) and sputtering (10 Pa).

### 3.2. Compositional, Morphological and Structural Analysis 

Energy dispersive X-ray spectroscopy (EDX) analysis confirmed the formation of five different NiFe compositions by varying the power applied to the Ni target (Supplementary Materials Table S1). Based on the atomic percentages of Fe, samples were named as Fe_20_Ni_80_, Fe_24_Ni_76_, Fe_28_Ni_72_, Fe_36_Ni_64,_ and Fe_38_Ni_62_. Increasing the Fe content usually improves the OER performance; however, beyond a certain threshold, Fe atoms tend to overpopulate, and the synergistic effect decreases. Effective arrangement of active sites is important to achieve maximum synergistic effect. Each synthesis process results in a different arrangement of the active sites; thus, the optimum Fe concentration varies, reported values ranging from 20% [38], 23% [11], 25% [39], 30% [40], and even 40% [27]. Thus, a wide range of 20–40% Fe content was chosen to identify the optimum composition for thin films synthesized through magnetron sputtering.

**Figure 1:**
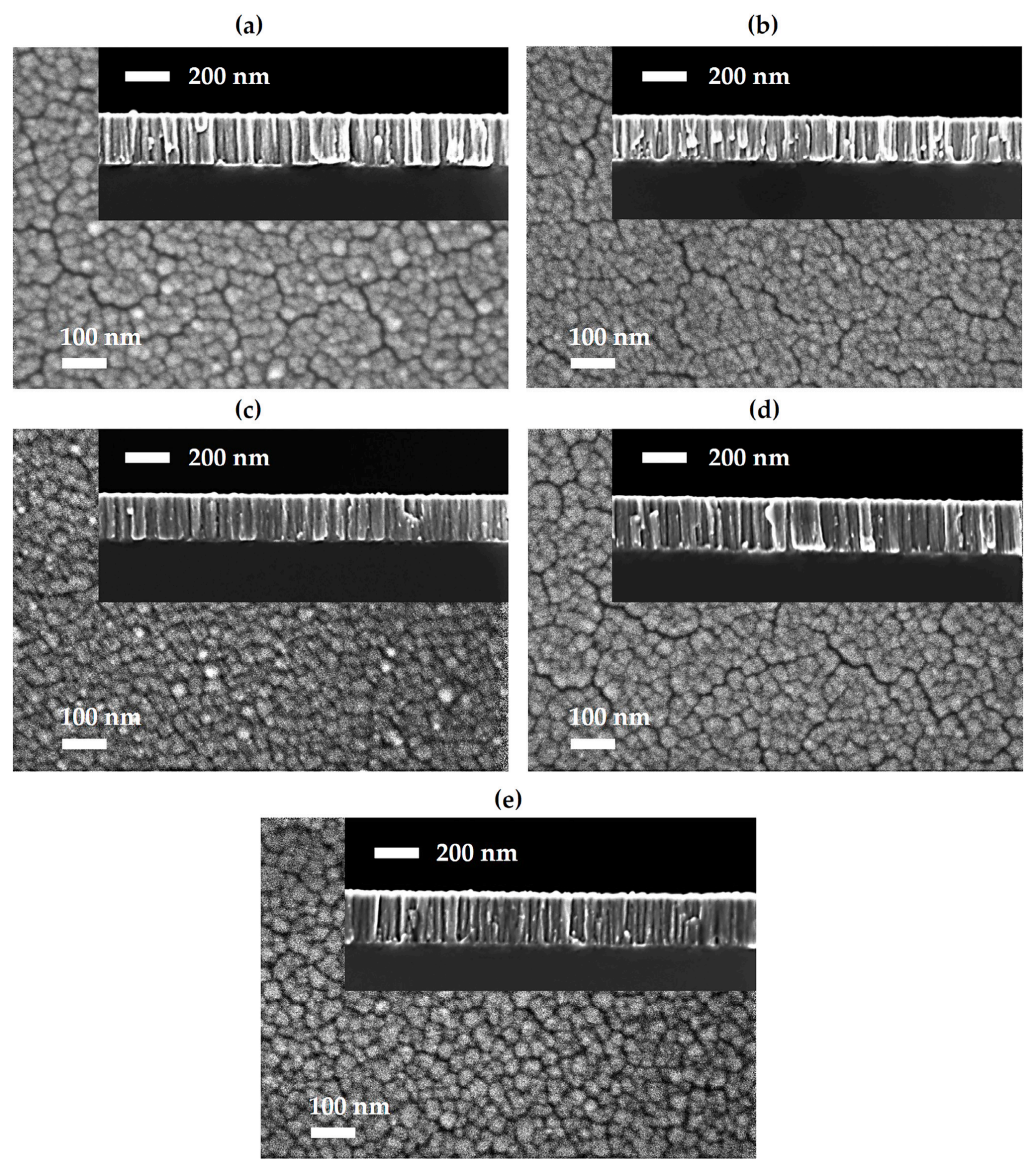
SEM images showing columnar morphology from the surface and cross-section (inset): (**a**) Fe_20_Ni_80_, (**b**) Fe_24_Ni_76_, (**c**) Fe_28_Ni_72_, (**d**) Fe_36_Ni_64_, (**e**) Fe_38_Ni_62_.

**Figure 2:**
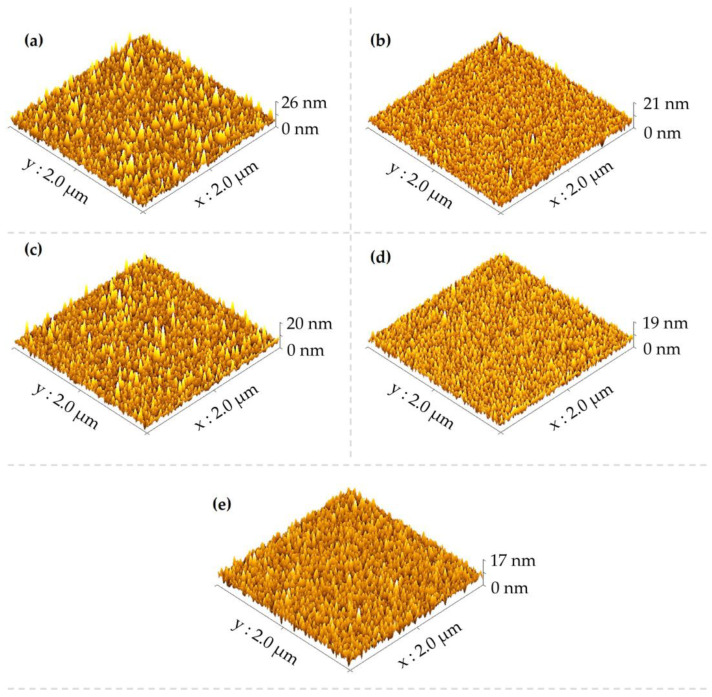
AFM images showing columnar morphology on the surface: (**a**) Fe_20_Ni_80_, (**b**) Fe_24_Ni_76_, (**c**) Fe_28_Ni_72_, (**d**) Fe_36_Ni_64_, (**e**) Fe_38_Ni_62_.

SEM images of thin films are shown in Figure 1a–e, where the main images show the surface and corresponding inset images show the cross-section. The morphology of all five thin films was similar. Cross-sectional images show a columnar morphology, typical of films grown by magnetron sputtering, and allow confirmation of the thickness of the films around 200 nm. Grains seen in the surface images are the top view of the columnar structure. Although grain size and column size seem to be similar, the surface of Fe_24_Ni_76_, Fe_36_Ni_64,_ and Fe_38_Ni_62_ samples shows what looks like “cracks”, which could suggest a more “open” morphology. However, Atomic Force Microscopy (AFM) images shown in Figure 2 confirmed that there were no cracks or open morphology; all five thin films had a similar columnar structure but had insignificant differences of up to 10 nm in the height of the columns. Surface roughness was measured through AFM, and mean surface roughness values (Table 1) were in a narrow range. This suggests that the morphology of these coatings does not play a significant role in explaining the electrochemical behavior observed later.

The interaction of the electrolyte with the electrode surface is affected by the hydrophobic/aerophilic nature of the surface. Wettability and air bubble tests were performed (Supplementary Materials Table S2) to check the nature of thin film depositions, and surface energies were also measured. The surface energy values presented in Table 1 show that all five thin-film depositions fall within a narrow range, except for the Fe_24_Ni_76_ sample, which exhibits a nearly 10% lower surface energy. Surface energy is dependent on composition, since other morphological factors are the same; thus, any difference in the electrochemical performance of the films could not be attributed to morphological factors and should be a function of composition only.

**Figure 3:**
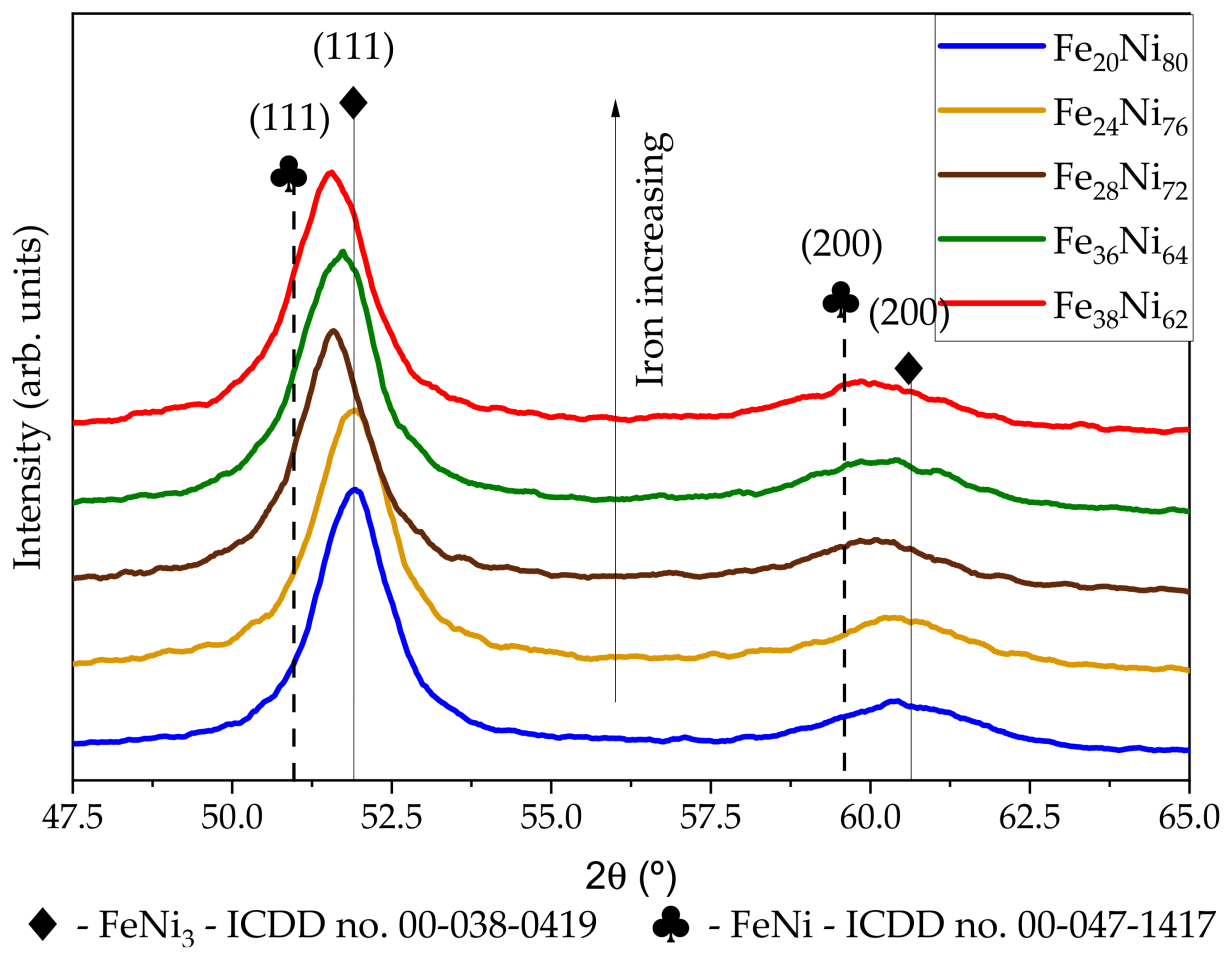
XRD Diffractograms.

Different crystallographic phases have different atomic densities, and this could lead to differences in electrochemical performance. XRD diffractograms of thin film depositions are shown in Figure 3. According to the Fe-Ni phase diagram [46,47], Fe_20_Ni_80_, Fe_24_Ni_76_ and Fe_28_Ni_72_ samples with Ni content higher than 70 atomic percent were expected to have pure FeNi_3_ phases, while Fe_36_Ni_64_ and Fe_38_Ni_62_ were expected to have α-Fe and FeNi_3_ phases. However, the two detected peaks do not match FeNi (ICDD no. 00-047-1417) or FeNi_3_ (ICDD no. 00-038-0419).

Broad shapes of the peaks and the proximity of the peak positions to Ni and Fe metal peaks suggest that the thin films were random solid solutions of Fe in Ni. There is a minor shift in peak position, where with increasing Fe content, XRD diffractograms showed a gradual shift towards smaller angles. This shift does not represent a difference in phase; rather, it is due to the difference in atomic radii of Fe and Ni. Fe has an atomic radius of 126 pm, and Ni has an atomic radius of 124 pm. When replacing one atom with the other in a random solid solution in the lattice, the slight difference in their atomic radii shall cause slight changes in the lattice parameters; thus, according to Bragg’s equation, peaks shall be shifted towards the smaller angles [48,49]. This peak shift hints towards the formation of random solid solutions instead of distinct phases. Conclusion about the formation of random solid solutions is further supported by Vegard’s law, which states that the lattice parameter of a solid solution varies linearly with the composition of the solid solution [50]. XRD diffractograms were analyzed and considering the formation of FCC (111) random solid solution, lattice parameters were calculated, and when plotted against composition, they showed a linear trend, thus further indicating the formation of a random solid solution (Supplementary Materials Section S3). Little deviations from the ideal linear behavior can be explained based on the magnetic nature of Ni and Fe and the inherent defects in the sputtered materials.

### 3.3. Electrochemical Analysis 

**Figure 4:**
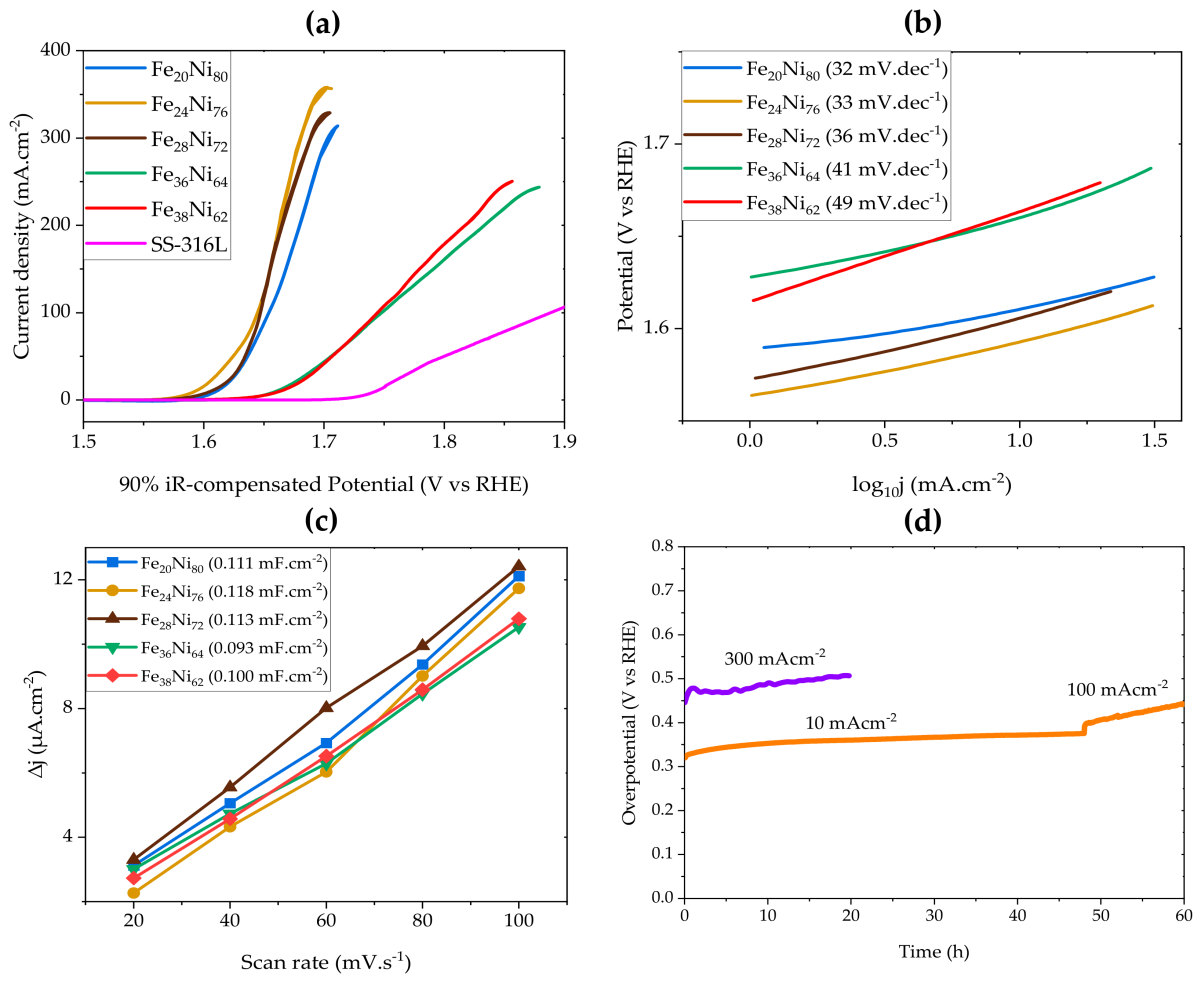
Electrochemical testing results: (**a**) 90% iR-compensated LSV scans, (**b**) Tafel plot, (**c**) double layer capacitance, (**d**) chronopotentiometry tests performed at 10 mA · cm^−2^, 100 mA · cm^−2^ and 300 mA · cm^−2^.

Electrochemical characterizations were performed to check the effectiveness of thin films as electro-catalyzing electrodes. LSV scans with 90% iR-compensation are shown in Figure 4a. At 10 mA · cm^−2^ and 100 mA · cm^−2^ current densities, Fe_24_Ni_76_ (361 and 414 mV, respectively) and Fe_28_Ni_72_ (373 and 418 mV, respectively) showed the lowest overpotentials versus RHE. OER overpotentials for all the coatings are given in Table 2.

An efficient OER-catalyzing electrode should provide large current densities at low operating potentials [33,34,51]. LSV scans showed that the synthesized thin-film anodes afforded large current densities. Under the mentioned LSV testing conditions, Fe_24_Ni_76_ achieved the largest current density of 363 mA · cm^−2^, and Fe_28_Ni_72_ was the next with 334 mA · cm^−2^ at the operating potential of 1.7 V vs. RHE.

Tafel slope represents the amount of voltage required to increase a decade of current. Hence, lower slopes represent better OER performance. Figure 4b shows the Tafel plot, with their respective slope values. Fe_20_Ni_80_, Fe_24_Ni_76_ and Fe_28_Ni_72_ compositions have marginally close Tafel slopes, so the kinetics and the rate-determining step shall be the same. However, the Fe_24_Ni_76_ composition has the lowest onset potential; thus, the OER shall be most easily initiated in this composition.

Figure 4c shows the plot of Δj (j_a_ − j_c_) against the scan rates in the non-Faradic region, yielding a slope for each line, which represents the double layer capacitance (C_dl_) [52,53]. Table 2 exhibits the C_dl_, RF, and ECSA values for the obtained thin film depositions, where Fe_24_Ni_76_ shows better property indicator values with C_dl_ 0.118 mF · cm^−2^, RF 2.95 and ECSA 10.53 cm^2^.

Chronopotentiometry tests confirmed the stable electrochemical performance of Fe_24_Ni_76_ under steady-state conditions. A constant current density of 10 mA · cm^−2^ was maintained for 48 h and then an increased current density of 100 mA · cm^−2^ was maintained for 12 h. Fe_24_Ni_76_ thin-film electrode showed a stable response over 60 h with no significant increase in required overpotential. Fe_24_Ni_76_ had a stable response even at a large current density, where a current density of 300 mA · cm^−2^ was maintained for 20 h (see Figure 4d).

**Figure 5:**
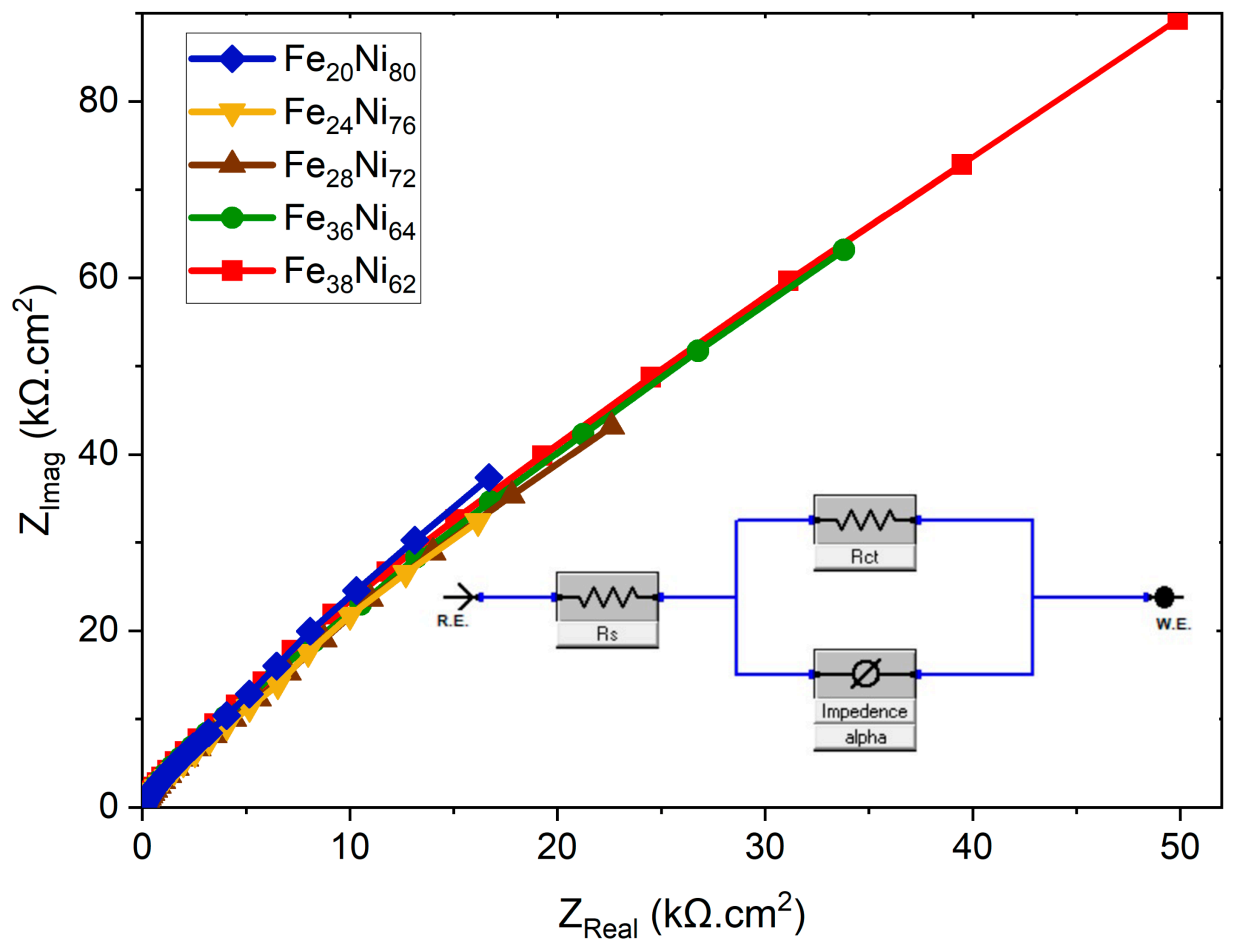
Nyquist plots and EEC.

Figure 5 shows the Nyquist plot obtained from EIS. Data fitting was performed over the Nyquist plots. Different equivalent electrical circuit (EEC) models were tested to fit the data, and the goodness of fit factor was assessed based on the chi-square (χ2) parameter, which should be within the range of 10^−4^ to 10^−3^ to be considered a good fit; lower values are an underfit and higher values are an overfit [54,55,56]. Figure 5 shows the EEC, which provided the best fit to the Nyquist plot. Here, R_s_ represents the solution resistance, R_ct_ is the charge transfer resistance, and Q is a constant phase element, which indicates the heterogeneous nature of the electrode surface [54,57]. The fitting parameters are shown in Table 3. Fe_24_Ni_76_ shows a comparatively lower R_ct_ value (14.91 kΩ), which means faster kinetics for OER catalysis [57]. Pertinent to mention that the absence of a well-defined semicircle further suggests that the fitted resistance does not represent true charge-transfer processes but likely reflects a combination of charge-transfer resistance, substrate passivation, incomplete catalyst activation, and limitations of the equivalent circuit model. Since the apparatus and all other testing parameters were the same, the values of R_ct_ can be used as comparative values.

Values of the alpha (α) coefficient (Table 3) from CPE were higher than 0.9, representing that the coatings behaved as capacitors, with no indication of corrosion/dissolution during the OER [58].

Electrochemical characterizations showed that thin film electrodes developed through DC magnetron sputtering can be used as efficient OER electrocatalysts. Among the five thin-film compositions, based on all the electrochemical parameters, Fe_24_Ni_76_ can be regarded as the optimum composition. Earlier studies on the OER performance of the NiFe alloys, using different synthesis processes, have also reported an optimum atomic percentage of Fe between 20 and 25 at.% [11,38,39].

### 3.4. OER Performance and the Composition of NiFe Solid Solution

Electrochemical performance of electrode material is affected by several structural, morphological and compositional factors. Structural and morphological factors were nearly constant and XRD indicates the formation of a random solid solution; thus, better performance of Fe_24_Ni_76_ shall be an outcome of the synergistic effect caused by bimetallic composition. In bimetallic or doped systems, an atom affects the neighboring atoms of the other species, thus creating a favorable synergy [3,59,60]. Li et al. [49] optimized the performance of an OER-catalyzing electrode by adjusting only the atomic percentage of Ni and Fe, as different properties are based on composition. In the present study, the best-performing Fe_24_Ni_76_ thin-film depositions had roughly 10% lower surface energy and comparatively lower charge transfer resistance, both of which are functions of composition.

XPS was performed over the best-performing Fe_24_Ni_76_ and the worst-performing Fe_38_Ni_62_ samples to check the difference in bonding states under as-deposited and OER-activated forms. XPS spectra were normalized to minimize the effect of compositional variations, as the analysis primarily focused on differences in bonding states.

High-resolution spectra were acquired for the Fe 2p and Fe 3p regions, but the Fe 3p region was used for the quantification of iron because the Fe 2p region overlaps with the Auger electron peaks Ni KLL, which were strong in these samples. Similarly, for nickel, Ni 2p and Ni 3p regions were acquired, but the Ni 3p region was used for quantification to obtain information from both Ni and Fe that is comparable. Indeed, Fe 3p and Ni 3p regions probe deeper into the surface of the sample, while Fe 2p and Ni 2p regions probe a shallower surface because of being at higher binding energy (BE) or lower kinetic energy.

**Figure 6:**
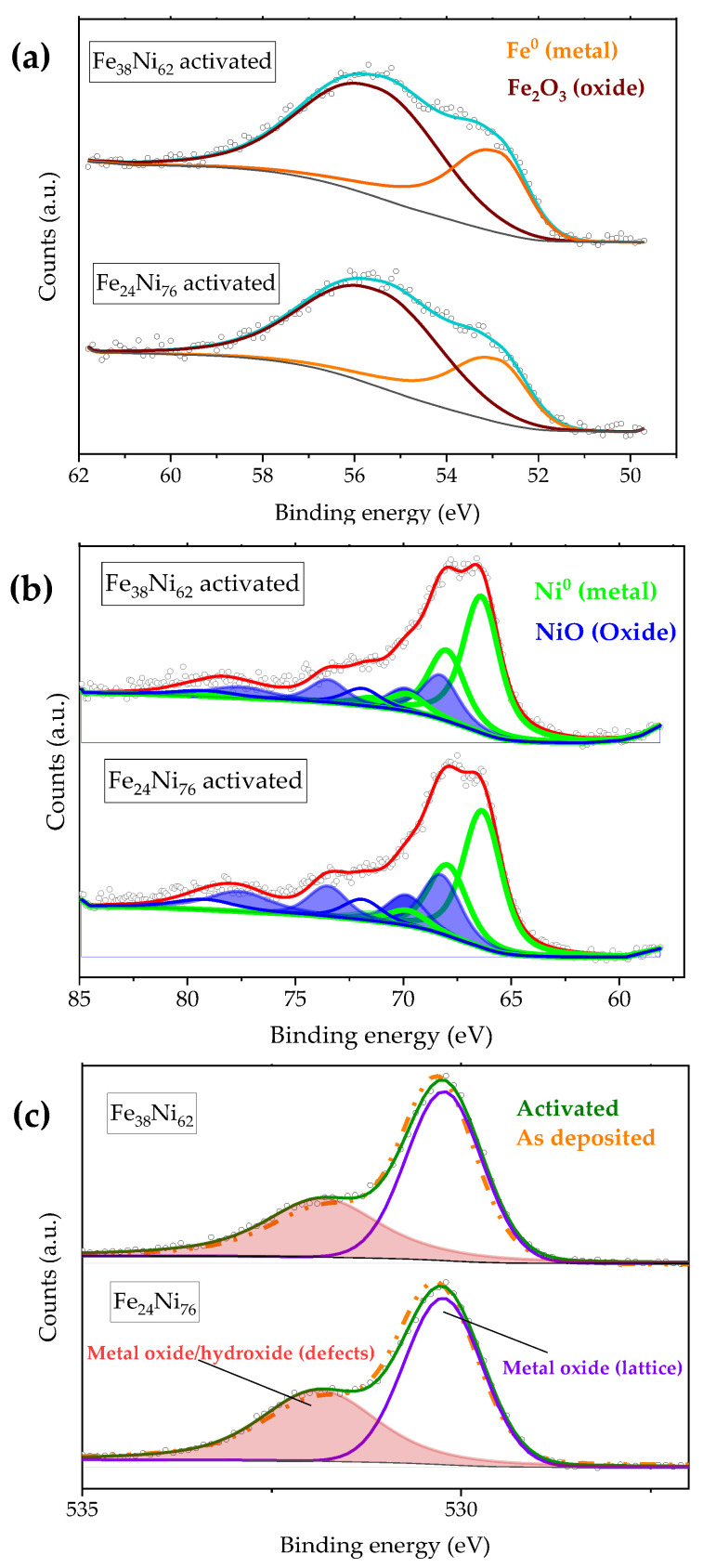
XPS spectra: (**a**) Fe 3p scan, (**b**) Ni 3p scan, (**c**) O 1s scan.

Fe 3p has a low spin–orbit coupling constant; thus, it can be treated as a single band without resolving it into Fe 3p_1/2_ and Fe 3p_3/2_ doublet [61]. Peak fitting of Fe 3p (Figure 6a) was performed with two asymmetric components for Fe^0^ (metal) at BE = 53.0 eV, and Fe_2_O_3_ (oxide) at BE = 55.6 eV, following the work of Yamahsita and Hayes [62].

Peak fitting of the Ni 3p region (Figure 6b) was performed using Ni 3p_3/2_ and Ni 3p_1/2_ doublets, with spin–orbit splitting = 1.6 eV and intensity ratio of Ni 3p_3/2_ to Ni 3p_1/2_ of 2. Comparing Ni 3p spectra from databases [63], the main (lowest BE) Ni 3p_3/2_ peak at BE = 66.4 eV was identified as Ni^0^ (metal) and the one at BE = 68.3 eV was identified as NiO (oxide).

O 1s (Figure 6c) was fitted with 2 component peaks. The lower BE component at BE = 530.0 eV is related to oxygen in metal oxides in the lattice. The component at BE = 531.9 eV can be related to defective metal oxide or metal hydroxide.

Differences in the atomic percentages of different components have been summarized in Table 4. Relative atomic percentages were calculated from the area of the peaks using manufacturer sensitivity factors.

XPS results in Table 4 show that, while Fe^0^ oxidation was similar in both samples, the Ni^0^ oxidation trend differed significantly between them. In the best sample (Fe_24_Ni_76_), a significant percentage of Ni^0^ metal got oxidized, while for the Fe_38_Ni_62_ sample, Ni^0^ metal oxidation was not so profound. Reaction mechanism of OER under alkaline medium [64] shows that metal oxides are formed as reaction intermediates during OER; thus, metal oxides/hydroxides also represent active reaction sites. Fe_24_Ni_76_ had a higher number of active sites because of the optimum synergistic effect; thus, it had better OER performance. This conclusion about the higher number of active sites is also supported by the highest value of double-layer capacitance for the Fe_24_Ni_76_ sample.

OER has a multistep mechanism involving adsorption/desorption steps [64]. Fe sites have strong adsorption for the OER intermediates, while Ni sites have weak adsorption, both not favorable for OER. Introduction of Fe in Ni creates a synergistic/coupling effect through local electronic coordination where the adsorption/desorption of Fe and surrounding Ni sites is optimized [29]. Thus, the bimetallic system becomes more OER efficient compared to its monometallic counterparts. Synergistic/coupling effect increases with an increase in Fe content up to an optimum composition. Beyond the optimum composition, the coupling effect weakens, and Fe sites become dominant on the catalyst surface. Due to the strong adsorption to the Fe sites, the surface gets covered with adsorbed intermediates and the OER is hindered [29,49].

More metal sites on the Fe_24_Ni_76_ sample were active for OER because its optimal NiFe composition and structure might influence the electronic state [27,65], reduce charge transfer resistance [27,64] and, subsequently, lower the required overpotential [27], explaining the promising electrochemical behavior of this sample.

## 4. Conclusions

In the present study, DC magnetron sputtering was used to successfully deposit NiFe thin films to be used as OER-catalyzing electrodes. Five different compositions, with increasing Fe content, were deposited and analyzed for optimum electrocatalytic performance.

Across all five compositions, SEM and AFM analyses revealed similar columnar morphologies and closely matched surface roughness values (RMS ≈ 1.1–1.8 nm). Wettability and surface energy measurements were likewise clustered in a narrow range for most coatings. Structural and morphological characterizations showed consistency and repeatability in all compositions of thin-film depositions. XRD analysis showed that all five compositions formed NiFe random solid solutions; thus, the Fe atoms were well dispersed in the Ni atoms.

Electrochemical characterizations confirmed the possibility of using thin films as OER-catalyzing surfaces. Among the tested compositions, Fe_24_Ni_76_ delivered the most promising performance, requiring only 361 mV overpotential to reach the current density of 10 mA · cm^−2^ and sustaining high current densities up to 363 mA · cm^−2^ at 1.7 V vs. RHE. Chronoamperometry long-term stability tests further confirmed the durability of Fe_24_Ni_76_ under sustained operation at moderate and high current densities.

The consistency of the film morphology and roughness across samples, together with electrochemical and XPS evidence, points to the decisive role of composition: Fe_24_Ni_76_ having an optimal Ni–Fe ratio and possibly having the most favorable local electronic coordination, thus having enhanced electronic structure, reduced charge-transfer resistance, and increased the concentration of active metal-oxide/hydroxide sites, thereby improving OER performance.

These promising results validate DC magnetron sputtering as a key technique to synthesize NiFe bimetallic electrodes in the form of thin films with controlled structure and effective arrangement of active sites in the form of a random solid solution. With targeted selection and optimization of substrate conductivity and thin-film architecture, sputtered NiFe thin-film electrodes may achieve industrial-level current densities (>500 mA · cm^−2^) while also having considerably lower costs, advancing scalable and sustainable hydrogen production.

## Figures and Tables

**Table 1:** Thin film characteristics.

Sr #	Sample Name	Film Thickness	Mean Roughness (RMS)	Surface Energy
		nm	nm	mN · m^−1^
1	Fe_20_Ni_80_	215 ± 3	1.75 ± 0.11	32.0
2	Fe_24_Ni_76_	205 ± 4	1.10 ± 0.10	29.7
3	Fe_28_Ni_72_	218 ± 4	1.24 ± 0.06	33.1
4	Fe_36_Ni_64_	209 ± 9	1.07 ± 0.09	32.8
5	Fe_38_Ni_62_	222 ± 5	1.28 ± 0.04	33.3

**Table 2:** Electrochemical characteristics.

Sr. #	Sample	Over Potential (mV)		Max Achieved Current Density (mA · cm^−2^)	Tafel Slope (mV · dec^−1^)	C_dl_ (mF · cm^−2^)	RF	ECSA cm^2^
		10 (mA · cm^−2^)	100 (mA · cm^−2^)					
1	Fe_20_Ni_80_	380	424	320	32	0.111	2.78	9.92
2	Fe_24_Ni_76_	361	414	363	33	0.118	2.95	10.53
3	Fe_28_Ni_72_	373	418	334	36	0.113	2.82	10.07
4	Fe_36_Ni_64_	439	518	243	41	0.093	2.32	8.28
5	Fe_38_Ni_62_	437	514	250	49	0.100	2.50	8.93

**Table 3:** EIS fitting parameters.

Sr. #	Sample	χ2 ×10^−4^	R_s_ Ω	R_ct_ kΩ	Q S.s^α^ ×10^−4^	α
1	Fe_20_Ni_80_	8.42	11	22.57	1.2	0.90
2	Fe_24_Ni_76_	2.65	8	14.91	1.7	0.91
3	Fe_28_Ni_72_	4.95	8	19.64	2.5	0.90
4	Fe_36_Ni_64_	2.93	10	26.54	3.3	0.92
5	Fe_38_Ni_62_	8.07	10	40.15	3.2	0.91

**Table 4:** Atomic percentages calculated from XPS analysis.

	Fe_24_Ni_76_		Fe_38_Ni_62_	
	As Deposited	Activated	As Deposited	Activated
Fe^0^ (Metal)	4.5	3.5	7.1	6.2
Fe_2_O_3_ (Oxide)	8.3	8.5	13.8	13.2
Ni^0^ (Metal)	26.5	22.8	19.4	20.6
NiO (Oxide)	16.4	17.2	11.1	10.5
O Metal oxides (lattice)	26.5	27.3	30.5	29.6
O Metal oxides/hydroxide (defects)	17.8	20.6	18.1	19.9
Fe_2_O_3_/Fe^0^ (Ratio of oxide to metal)	1.84	2.43	1.94	2.13
NiO/Ni^0^ (Ratio of oxide to metal)	0.62	0.75	0.57	0.51

## Data Availability

The original contributions presented in the study are included in the article/Supplementary Materials, further inquiries can be directed to the corresponding author.

## Supplementary Materials

The following supporting information can be downloaded at: https://www.mdpi.com/article/10.3390/ma19153155/s1, Section S1: Deposition parameters; Section S2: Wettability and air bubble test; Section S3: XRD analysis for verification of Vegard’s rule; Table S1: Deposition parameters; Table S2: Wettability measurements; Table S3: Lattice parameter calculation; Figure S1: Plot of lattice parameter against composition. Reference [66] is cited in the Supplementary Materials.
