# Supplementary material for: DC Magnetron Sputtering for Synthesizing Bimetallic NiFe Thin Films as Efficient OER-Catalyzing Electrodes
Source: Materials (Basel). 2026 Jul 23;19(15):3155. doi: 10.3390/ma19153155 (PMC13466277; doi:10.3390/ma19153155)
Supplement: Supplementary file 1 [file materials-19-03155-s001.zip › materials-4427311-supplementary.pdf]

SUPPLEMENTARY DATA

S1. DEPOSITION PARAMETERS

Sr #	Sputtering power (W)		Deposition time	Composition		Sample name
	Invar	Ni	min:s	Fe	Ni	
**1**	1000	1200	3.30	20.0 ± 0.8	80.0 ± 0.8	$Fe_{20}Ni_{80}$
**2**	1000	1000	4.00	24.0 ± 0.4	76.0 ± 0.2	$Fe_{24}Ni_{76}$
**3**	1000	800	4.30	28.0 ± 0.7	72.0 ± 0.7	$Fe_{28}Ni_{72}$
**4**	1000	600	5.30	36.0 ± 0.2	64.0 ± 0.1	$Fe_{36}Ni_{64}$
**5**	1000	500	6.00	38.0 ± 0.4	62.0 ± 0.4	$Fe_{38}Ni_{62}$

Table S1: Deposition parameters

S2. WETTABILITY AND AIR BUBBLE TEST

S2.1. Procedure

S2.1.1. Sessile drop method

The wettability of NiFe thin films deposited over an SS substrate was assessed using the sessile drop method with an Attension® Theta Flex (Biolin Scientific) contact angle system.

S2.1.2. Captive bubble method

To assess the aerophobicity of the coatings, the captive bubble method was applied. Coated samples (1 × 1 cm) were affixed in an inverted position onto a glass substrate and submerged in distilled water. An air bubble was introduced underneath the sample, and the contact angle was measured using the sessile drop configuration.

S2.1.3. Surface energy calculation

Distilled water, diiodomethane, and bromonaphthalene were used to determine the surface free energy (SFE) by calculating the polar (γ^p) and non-polar (γ^d) components using the Owens-Wendt-Rabel-Kaelble (OWRK) method, following the international standard ISO 19403-2. [1]

Reference

1. D.K. Owens, R.C. Wendt, Estimation of the surface free energy of polymers, J Appl Polym Sci 13 (1969) 1741–1747. https://doi.org/10.1002/APP.1969.070130815.

S2.2. Contact Angle measurements

		$Fe_{20}Ni_{80}$	$Fe_{24}Ni_{76}$	$Fe_{28}Ni_{72}$	$Fe_{36}Ni_{64}$	$Fe_{38}Ni_{62}$
**Surface Energy**	**Y tot (mN/m)**	32	29.7	33.1	32.8	33.3
	**Y d (mN/m)**	32	29.6	33	32.5	32.8
	**Y p (mN/m)**	0	0.2	0.1	0.3	0.5
**Contact angle Sessile drop method**	**Water**	106	103	101	99	98
	**Diiodomethane**	45	57	51	53	55
	**Bromonaphthalene**	55	52	45	44	41
**Contact angle Captive bubble method**	**Water**	106	103	101	99	98
	**Diiodomethane**	45	57	51	53	55
	**Bromonaphthalene**	55	52	45	44	41

Table S2: Wettability measurements

S3. XRD ANALYSIS FOR VERIFICATION OF VEGARD'S RULE

Samples	2θ (°)	θ (Radians)	λ (nm) for Co Kα	d-spacing Å	a (111) Å
**Pure Ni (FCC)**					3.520
**$Fe_{20}Ni_{80}$**	51.89	0.452852	0.179	2.045567	3.543
**$Fe_{24}Ni_{76}$**	51.89	0.4528	0.179	2.045786	3.544
**$Fe_{28}Ni_{72}$**	51.59	0.450216	0.179	2.056716	3.561
**$Fe_{36}Ni_{64}$**	51.66	0.450783	0.179	2.054305	3.563
**$Fe_{38}Ni_{62}$**	51.56	0.449946	0.179	2.057864	3.565
**Pure Fe (FCC)**					3.650

Table S3: Lattice parameter calculation

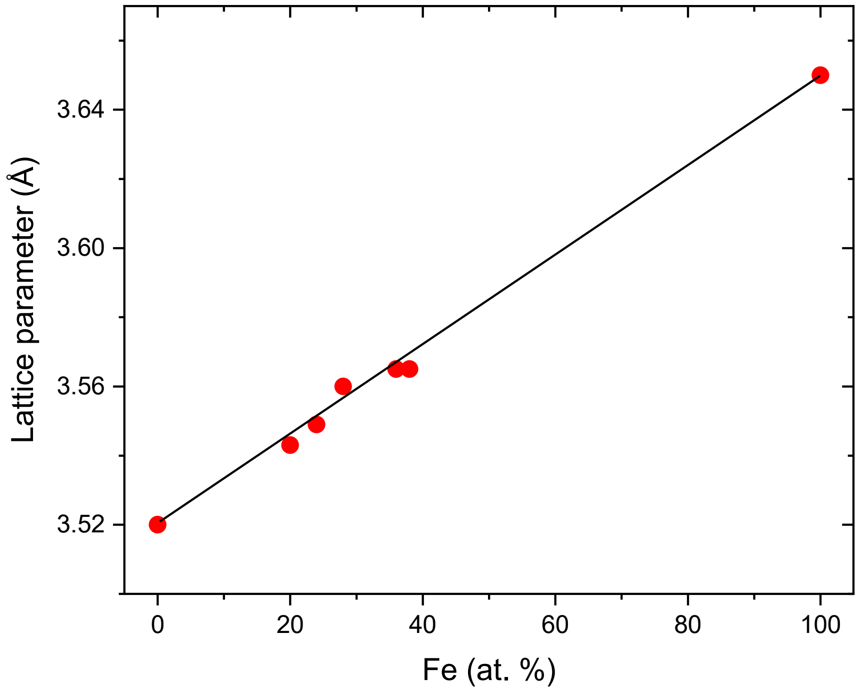

Figure S1: Plot of lattice parameter against composition
